# Dermoscopy of Onychopapilloma: A Benign Mimic of Subungual Malignancy

**DOI:** 10.7759/cureus.78103

**Published:** 2025-01-27

**Authors:** Brad R Woodie, Sweta Subhadarshani

**Affiliations:** 1 Department of Dermatology, University of Cincinnati College of Medicine, Cincinnati, USA; 2 Department of Dermatology, Perelman School of Medicine, University of Pennsylvania, Philadelphia, USA

**Keywords:** longitudinal erythronychia, nail bed tumor, nail biopsy, nail dermoscopy, nail diseases, nail matrix tumor, onychopapilloma

## Abstract

Onychopapilloma is a benign tumor of the nail bed and distal matrix, commonly presenting with longitudinal erythronychia and distal subungual hyperkeratosis. A 30-year-old Asian male presented with an asymptomatic, stable discoloration of the thumbnail, with longitudinal erythronychia, distal onycholysis, and V-shaped fissuring. Dermoscopy revealed splinter hemorrhages and focal distal subungual hyperkeratosis beneath the V-shaped notch. The lesion was unchanging over several years, and the patient opted for conservative management with periodic monitoring rather than biopsy. Based on clinical and dermoscopic features, onychopapilloma can often be distinguished from malignant conditions such as amelanotic melanoma and squamous cell carcinoma. Differential diagnoses include glomus tumor, trauma, Darier disease, and lichen planus. While biopsy is not needed for stable, asymptomatic cases, it is recommended if the lesion changes or becomes symptomatic.

## Introduction

Onychopapilloma is a benign tumor of the nail bed and distal matrix, typically affecting the thumb, and classically presents with longitudinal erythronychia and distal subungual hyperkeratosis [1]. First described in 1995 as localized multinucleate distal subungual keratosis [2], onychopapilloma can also present with longitudinal leukonychia, longitudinal melanonychia, splinter hemorrhages, and yellow-brown chromonychia [3,4]. Nail abnormalities may include nail fissuring, a distal V-shaped notch, and onycholysis [3]. Onychopapilloma may mimic malignancies such as melanoma and squamous cell carcinoma, so awareness of distinguishing features can help reassure patients and avoid unnecessary biopsies. We report a case of onychopapilloma in which the characteristic elements were identified with the help of dermoscopy.

## Case presentation

**Figure 1:**
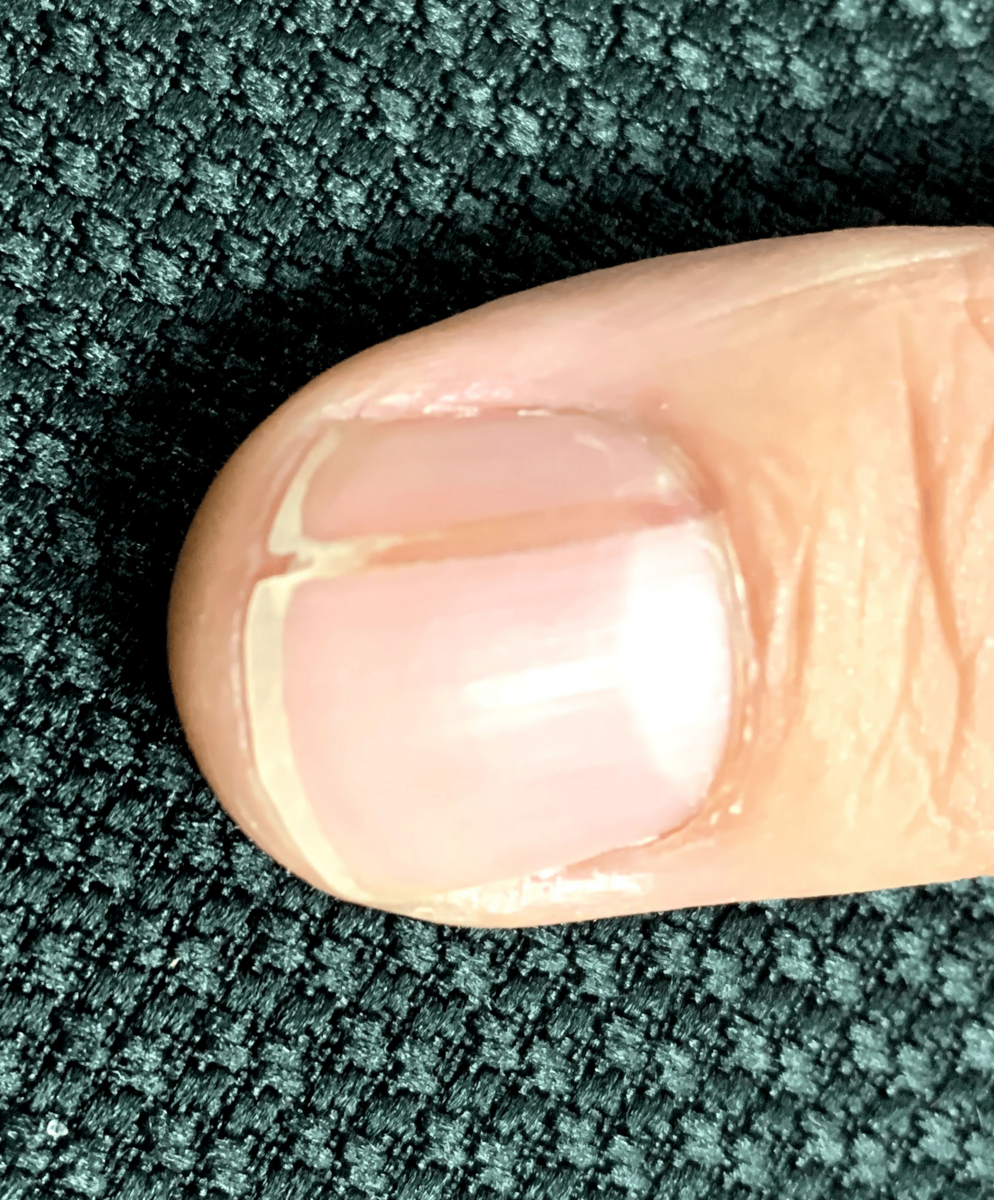
Clinical presentation of a single thumbnail with longitudinal erythronychia, distal V-shaped fissuring, and onycholysis

A 30-year-old Asian male presented with a many-year history of unchanging and asymptomatic discoloration affecting a single thumbnail. The patient denied a history of local trauma to the area and did not recall the initial onset. Upon physical examination, the nail exhibited longitudinal erythronychia with distal onycholysis and V-shaped fissuring (Figure 1).

**Figure 2:**
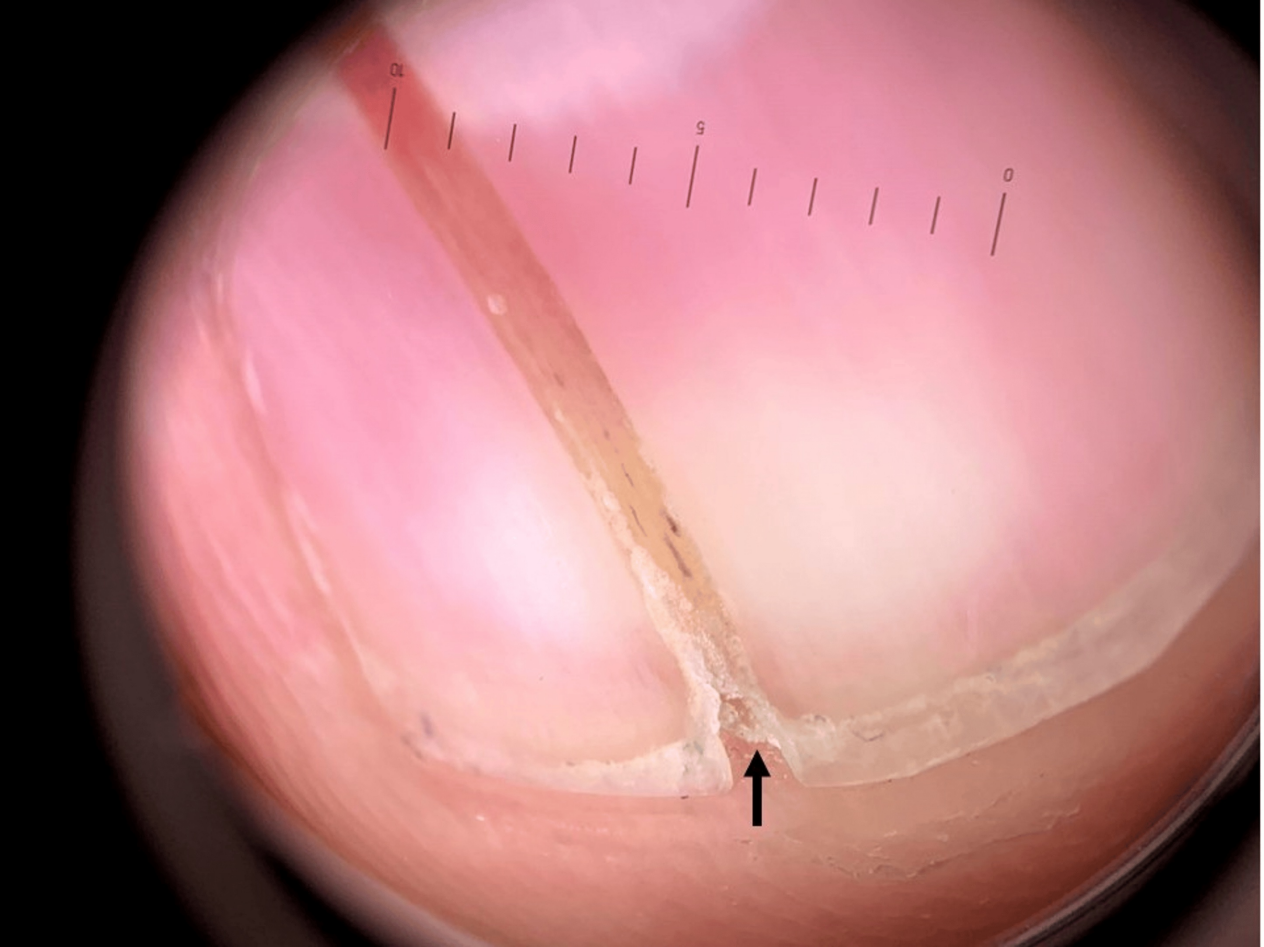
Dermoscopy reveals short splinter hemorrhages and focal distal subungual hyperkeratosis (arrow) beneath the V-shaped notch

There was no tenderness to palpation. The dermoscopic evaluation permitted the detection of short splinter hemorrhages within the band and revealed focal distal subungual hyperkeratosis beneath the V-shaped notch (Figure 2).

The rest of the mucocutaneous examination was unremarkable. A biopsy was not performed, as the patient preferred conservative management consisting of annual clinical and dermoscopic evaluations, with instructions to return sooner if any changes were observed.

## Discussion

Clinical and dermoscopic findings may enable the clinician to diagnose onychopapilloma without biopsy. Distal subungual hyperkeratosis, which may only be detected dermoscopically, helps distinguish onychopapilloma from subungual malignancies such as amelanotic melanoma and squamous cell carcinoma. The prevalence of subungual hyperkeratosis in onychopapilloma has varied between case series. A keratotic subungual mass was present in all cases reported by Tosti et al. [1], in 58% of cases by Yun et al. [5], and in 29% of cases by Starace et al. [3]. Dermoscopy can also permit visualization of a convex, pointed, or dome-shaped border in the lunula [1,3]. Erythronychia may blanch when pressure is applied, and dermoscopy can improve the detection of splinter hemorrhages [1].

In addition to melanoma and squamous cell carcinoma, the differential diagnosis of onychopapilloma includes glomus tumor, local trauma, Darier disease, and lichen planus. Glomus tumors can present with longitudinal erythronychia, but the affected digit typically shows a triad of tenderness, paroxysmal pain, and cold hypersensitivity [6]. Glomus tumors also usually appear as a bluish or purple subungual nodule and may have an associated distal keratotic mass [7,8]. A local injury should be considered, as a wooden splinter was recently reported to cause longitudinal erythronychia with distal subungual keratosis [9]. Most patients with Darier disease have nail involvement, and longitudinal erythronychia may have associated subungual hyperkeratotic papules [10]. However, multiple nails are affected and classically have alternating red and white stripes, referred to as “candy-cane nails” [11]. Nail bed lichen planus may initially present with longitudinal erythronychia of a single nail but can be distinguished from onychopapilloma with onychorrhexis and nail plate atrophy [7,12].

In the present case, histopathological diagnosis was not pursued because the lesion was asymptomatic and unchanging over many years. When the diagnosis is uncertain, classical longitudinal excision with longitudinal sectioning is the most accurate technique [13]. Malignant onychopapilloma has recently been reported [14], so tissue should be obtained if a lesion is evolving or symptomatic. In the absence of atypical features, stable onychopapilloma can be monitored with periodic measurement and dermoscopic evaluation [7]. Follow-up intervals may be individualized based on the characteristics of the suspected onychopapilloma and patient preferences, such as three months for a new lesion or annually for a stable lesion [7].

## Conclusions

In conclusion, onychopapilloma is a benign nail condition that can often be diagnosed based on characteristic clinical and dermoscopic findings, eliminating the need for biopsy in stable, asymptomatic cases. Dermoscopy plays a key role in distinguishing onychopapilloma from malignant lesions and other differential diagnoses. Regular monitoring is recommended for stable lesions, with biopsy considered if the lesion becomes symptomatic or evolves, as rare cases of malignant transformation have been reported. This case highlights the importance of a thorough clinical and dermoscopic evaluation in the management of onychopapilloma.
